# Differences in the Impact of Left Ventricular Outflow Tract Obstruction on Intraventricular Pressure Gradient in Feline Hypertrophic Cardiomyopathy

**DOI:** 10.3390/ani14223320

**Published:** 2024-11-19

**Authors:** Miki Hirose, Momoko Watanabe, Aki Takeuchi, Aimi Yokoi, Kazuyuki Terai, Katsuhiro Matsuura, Ken Takahashi, Ryou Tanaka

**Affiliations:** 1 Veterinary Teaching Hospital, Tokyo University of Agriculture and Technology, Fuchu 183-0054, Tokyo, Japan; s212240r@st.go.tuat.ac.jp (M.H.); fv5028@go.tuat.ac.jp (A.T.); aimi.y139@gmail.com (A.Y.); terai.kazuyuki.dvm@gmail.com (K.T.); 2 Animal Medical Centre Fanling, Po Hon Lau, 17 Luen On St., Fanling New Territories, Hong Kong, China; momokowatanabe526@gmail.com; 3 Department of Small Animal Clinical Sciences, College of Veterinary Medicine University of Florida, Gainesville, FL 32608, USA; kmatsuura@ufl.edu; 4 Department of Pediatrics and Adolescent Medicine, Juntendo University Graduate School of Medicine, Tokyo 113-8421, Japan; kentaka@juntendo.ac.jp

**Keywords:** diastolic function, intraventricular pressure gradient, color M-mode, cat, hypertrophic cardiomyopathy, left ventricular outflow tract obstruction, echocardiography

## Abstract

Hypertrophic cardiomyopathy (HCM) is a common form of cardiomyopathy in cats, and heart failure occurs as diastolic dysfunction progresses. HCM in cats is broadly classified as non-obstructive and obstructive hypertrophic cardiomyopathy, depending on the presence or absence of outflow tract obstruction. Measurement of the intraventricular pressure differences (IVPD) using color M-mode (CMM) has attracted attention as a reliable diastolic index as it correlates with catheterization, the gold standard for the assessment of diastolic performance. Because IVPD is affected by the size of the heart, the intraventricular pressure gradient (IVPG) index, which is unaffected by heart size, is by calculated by dividing IVPD by LV length. In the present study, CMM IVPG was used to non-invasively assess diastolic impairment in cats with obstructive hypertrophic cardiomyopathy. This study was conducted on 10 control cats and 18 cats in the HCM group. Although no severe left atrial enlargement was observed in the HCM group, the basal IVPG was significantly increased in the HOCM group compared to the control group. Although IVPD typically suggests impaired diastolic function and reduced ventricular compliance, the significant increase observed in the HOCM group compared to controls may suggest an indirect elevation in left atrial pressure, likely secondary to left ventricular outflow tract obstruction. The increase in IVPG in HOCM, as shown in this study, is a pathological effect of left ventricular outflow tract obstruction that cannot be detected by conventional echocardiographic indices, and evaluating IVPG is useful to evaluate cardiac function from a perspective that differs from conventional methods.

## Simple Summary

In recent years, intraventricular pressure gradient (IVPG) measured using color M-mode, which is excellent for evaluating diastolic function, has gained attention. In this study, the IVPG of cats with hypertrophic cardiomyopathy (HCM) was evaluated. The results showed that in non-obstructive HCM (HNCM), there were no changes in IVPG, whereas in hypertrophic obstructive cardiomyopathy (HOCM), IVPG increased despite a suspected decline in diastolic function. The fact that a significant difference was observed only with IVPG suggests the existence of pathophysiological differences that can only be detected by IVPG. By using IVPG for the evaluation of HOCM, it is expected that more accurate assessment of the condition, improvement of treatment methods, and new prognostic indicators, which were previously unattainable, will become possible.

## 1. Introduction

Hypertrophic cardiomyopathy (HCM) is a common cardiomyopathy in cats that predominantly impairs diastolic function, underscoring the need for effective diagnostic and treatment approaches [1]. Diastolic function, critical in HCM assessment, includes “active relaxation” during early diastole, when the left ventricle draws blood from the left atrium, and “passive relaxation” during late diastole. Measuring diastolic function traditionally requires catheterization, which is considered the gold standard, yet its invasiveness limits its application in veterinary practice. Conventional echocardiographic methods, including the E/A ratio and tissue Doppler imaging (TDI), have limitations in diastolic function assessment in cats, especially due to high heart rates that often fuse left ventricular inflow waveforms [2,3].

Recent advancements have highlighted the potential of color M-mode (CMM) echocardiography to non-invasively measure intraventricular pressure differences (IVPD), offering a simpler, rapid, and reliable approach for clinical use that correlates well with catheterization [4,5].

IVPD, which reflects the pressure gradient across the ventricle, varies with heart size, and thus the intraventricular pressure gradient (IVPG) was developed as a size-independent index by normalizing IVPD to the left ventricular length. Recent animal studies have shown segmental variations in IVPD, with basal IVPD increasing under volume load, whereas mid-to-apical IVPD decreases with declining diastolic function [6].

In clinical practice, HCM in cats is classified into hypertrophic obstructive (HOCM) and non-obstructive (HNCM) types, based on the presence of left ventricular outflow tract obstruction (LVOT). This obstruction, often due to myocardial hypertrophy or systolic anterior motion (SAM) of the mitral valve, leads to increased intraventricular pressure [7].

Given the prevalence of asymptomatic HCM in cats, which is associated with risks of heart failure and sudden death [8,9], improved non-invasive methods for assessing diastolic function are critical. In this study, we hypothesize that CMM-derived IVPD can offer a more detailed evaluation of diastolic dysfunction in HCM, providing insights into the different pathophysiological impacts of LVOT on HOCM and HNCM in feline cardiology.

## 2. Materials and Methods

### 2.1. Animals and Study Protocol

Between October 2021 and April 2023, 18 cats diagnosed with HCM and 10 control cats without heart disease were included in the study, which was conducted at a pediatric hospital for cats and dogs. Cats with a left ventricular end-diastolic wall thickness exceeding 6 mm as measured by M-mode echocardiography were classified as the HCM group.

In this study, HOCM was defined by the presence of either SAM of the mitral valve or a left ventricular outflow tract velocity (LVOTv) of 250 cm/s or higher. Cats with HCM but without SAM and with an LVOTv of 250 cm/s or below were classified into the HNCM group.

The control group consisted of healthy cats without any cardiovascular disease, as confirmed by echocardiography and electrocardiography. These cats were free from systemic conditions that could affect the cardiovascular system, such as hypertension, renal dysfunction, or endocrine disorders.

### 2.2. Conventional Echocardiography

Echocardiographic examinations were performed using an ultrasound machine (LISENDO880, Fujifilm, Tokyo, Japan). Echocardiographic images were stored offline and analyzed using commercial software (described later). The cats were positioned in lateral recumbency, and standard left and right cardiac windows were used to record conventional echocardiographic measurements.

From the right parasternal short-axis view at the level of the papillary muscles, the following measurements were reported: left ventricular internal diameter at end-diastole and end-systole (LVIDd, LVIDs), interventricular septal thickness at end-diastole and end-systole (IVSd, IVSs), left ventricular free wall thickness at end-diastole and end-systole (LVPWd, LVPWs), and fractional shortening (FS). From the right parasternal short-axis view at the base of the heart, the left atrial diameter (LAD), aortic diameter (Ao), and left atrium-to-aorta ratio (LA/Ao) were measured in two-dimensional echocardiography. At the level of the left ventricular apex, left ventricular outflow tract velocity (LVOTv) (five-chamber view) and mitral inflow velocity (Ev) (apical four-chamber view) were measured using pulsed-wave Doppler echocardiography. Using tissue Doppler imaging (TDI), early diastolic tissue velocity (e’) was measured from the left ventricular free wall (FW). The ratio of Ev to e’ (E/e’) was then calculated for the left ventricular free wall. All echocardiographic measurements were obtained over three cardiac cycles and averaged. LVPW, IVS, LVID, and FS were measured from the right parasternal short-axis view at the papillary muscle level using M-mode, with the leading-edge-to-leading-edge method. LA/Ao was measured in B-mode at the base of the heart in early diastole, just after the closure of the aortic valve.

Mitral inflow velocity was measured using pulsed-wave Doppler from the left apical four-chamber view, with the sample volume placed at the tips of the mitral valve leaflets. When the E wave and late peak wave (Av) could not be clearly distinguished, the waveform was classified as fused.

### 2.3. Analysis of Intraventricular Pressure Gradient (IVPG)

**Figure 1:**
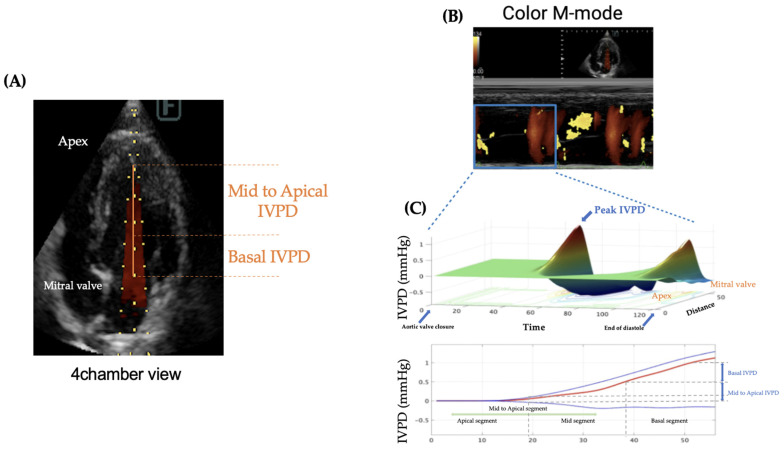
Intraventricular pressure difference measurement. First, the mitral inflow was optimized from the left apex four-chamber view. The CMM settings were then switched on to measure IVPD and the appropriate images were extracted (**A**). The extracted images were further processed in MATLAB software (version R2021b) to calculate IVPD and IVPG; the 3D profile of IVPD was calculated by extracting velocity, temporal and spatial components from the region of interest (blue box) (**B**). Spatial distribution of IVPD along the entire left ventricle from the base to the apex (**C**). Of the left ventricular length, one-third on the apex side was calculated as an apical segment, the middle one was calculated as a mid-segment, and one-third on the basal side of the heart was calculated as a basal segment. The top (blue), middle (red), and bottom (blue) lines represent inertial, total, and convective IVPD, respectively.

Figure 1 illustrates the process of IVPD measurement. All examinations were performed without sedation, and tests were temporarily halted in cases of rapid increases in heart rate or respiratory rate to avoid stress-induced artifacts. Because cats are particularly susceptible to stress, which can elevate their heart rate even from a visit to the hospital, the examination room was kept dim and quiet to minimize stress. The entire echocardiographic protocol, including CMM, was completed within 10 min. Ultrasound settings were adjusted, especially the gain, to appropriately visualize the entire mitral inflow tract and the LV apex from the left apical four-chamber view to trace the IVPD. Pre-settings included a sweep speed of 300 mm/s, color baseline shift of −64, and velocity range set to approximately 1.5 times the recorded mitral inflow velocity at the valve tips to raise the Nyquist limit for color Doppler imaging.

For the image processing of IVPD and IVPG using MATLAB, the time from aortic valve opening to closure, the time from the onset of the Q wave to the start of Ev, and the time from the onset of the Q wave to the peak of the E wave were collected from conventional echocardiography and manually input into a MATLAB dialog box as per the program instructions. Three high-quality images from consecutive heartbeats were selected for each case, and the average values for each patient were used in the analysis.

IVPD and IVPG from CMM were calculated based on a previously validated IVPD measurement method [4]. IVPD was computed from the images obtained by CMM and processed in MATLAB (The MathWorks, Natick, MA, USA) using the following Euler equation:

(∂P)/(∂s) = −ρ((∂v)/(∂t) + v(∂v)/(∂s))(1)

where ∂ represents changes in the followed element, P is pressure, ρ is the constant blood density (1060 kg/m^3^), v is velocity, s is the position along the color M-mode line, and t is time. IVPG values were derived from IVPD using the following formula [10]:

IVPG (mmHg/cm) = IVPD/LV length(2)

Both IVPG and IVPD were automatically divided into the basal, mid, and apical segments of the left ventricle (LV) by the program [11]. The mid-to-apical IVPG method involves analyzing the apical two-thirds of the left ventricle, a modification adopted to account for measurement challenges in small animals and to ensure stable values.

### 2.4. Inclusion and Exclusion Criteria

Only cases in which the color M-mode (CMM) image of the left ventricular apex could be clearly visualized were included in the analysis. Exclusion criteria consisted of cases with severe left ventricular outflow tract obstruction, significant turbulence that interfered with intraventricular pressure gradient analysis, or severe congestion that compromised image quality. Additionally, cats with systemic hypertension (systolic blood pressure ≥ 180 mmHg), hyperthyroidism, or secondary HCM due to significant dehydration were excluded.

### 2.5. Treatment Protocol

Of the 18 cats diagnosed with HCM, 13 were treated with beta-blockers (Artist tablets; Carvedilol, Daiichi Sankyo Co., Ltd., Tokyo, Japan, 0.05~0.4 mg/kg/day, oral route) (10 in stage B1, 1 in B2, and 1 in C), 4 were treated with angiotensin-converting enzyme inhibitors (Fortecor Tablets; Benazepril hydrochloride, Elanco Japan Co., Ltd., Tokyo, Japan, 2.5 mg/head, oral route, once a day) (3 in stage B1 and 1 in C), 3 were treated with antiplatelet agents (Plavix tablets 75 mg; Clopidogrel Sulfate, Sanofi K.K., Tokyo, Japan, 18.75 mg/head, oral route, once a day). One of the stage C cats with HCM was treated with anticoagulants (Xarelto Tablets; Rivaroxaban, Bayer Yakuhin Co., Ltd., Osaka, Japan, 2.5 mg/head, oral route, once a day), while another was treated with diuretics (Lasix tablets; Furosemide, Sanofi K.K., Tokyo, Japan, 1 mg/kg, oral route, twice a day).

### 2.6. Statistical Analysis

The statistical methods used included the Mann–Whitney test and the Kruskal–Wallis test. Simple linear regression was performed between traditional Ev and IVPG measurements. The interpretation of effect size was based on the rc value, with rc ≥ 0.5 considered effective. The statistical software used for the analysis was GraphPad Prism Version 8 (GraphPad Software Inc., San Diego, CA, USA), and comparisons were made across all echocardiographic data for the control, HNCM, and HOCM groups. In this study, a *p*-value of <0.05 was considered statistically significant for the analyzed variables.

## 3. Results

### 3.1. Patient Data

The most common breeds in the control group were American Shorthair and Maine Coon (*n* = 2), followed by Scottish Fold (*n* = 1), British Shorthair (*n* = 1), Siberian (*n* = 1), Abyssinian *(n* = 1), Selkirk Rex (*n* = 1), and Minuet (*n* = 1). The most common breeds in the HCM group were Scottish Fold and mixed breed (*n* = 5), followed by American Shorthair (*n* = 3), Munchkin (*n* = 2), Russian Blue (*n* = 1), American Curl (*n* = 1), and Persian (*n* = 1). The median age of the control group was 1 year (range: 0.25–9 years), and the median age of the HCM group was 4 years (range: 0.5–15 years); the median body weight of the control group was 2.95 kg (range: 1.65–5.1 kg), and the median body weight of the HCM group was 4.25 kg (range: 2.36–6.3 kg). The control group consisted of 6 males and 4 females, and the HCM group consisted of 12 males and 6 females.

### 3.2. Conventional Echocardiography Results

**Figure 2:**
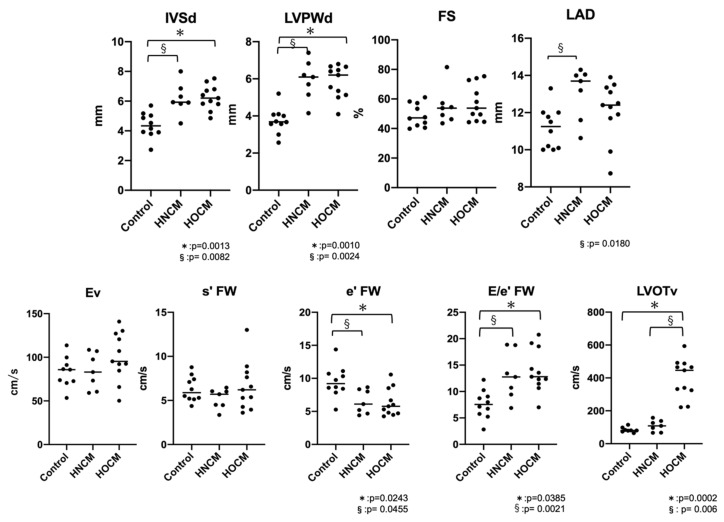
Conventional echocardiographic in the control (*n* = 10), HNCM (*n* = 7) and HOCM (*n* = 11) groups. Horizontal lines indicate median values. *, §, indicates statistical significance (*p* < 0.05). IVSd: interventricular septum thickness at end-diastole; LVPWd: left ventricular posterior wall thickness at end-diastole; FS: left ventricular fractional shortening; LAD: left atrial diameter; LVOTv: left ventricle outflow tract velocity; E: peak velocity of early diastolic transmitral flow velocity; s′: peak velocity of systolic mitral annular motion as determined by color-based tissue Doppler imaging; e′: peak velocity of early diastolic mitral annular motion as determined by color-based tissue Doppler imaging; FW: free wall; HNCM: hypertrophic non-obstructive cardiomyopathy; HOCM: hypertrophic obstructive cardiomyopathy.

The results of the conventional echocardiography are shown in Table 1 and Figure 2. IVSd and LVPWd were significantly increased in both the HOCM and HNCM groups compared to the control group (IVSd: control vs. HNCM *p* = 0.0082, control vs. HOCM *p* = 0.0013, LVPWd: control vs. HNCM *p* = 0.0024, control vs. HOCM *p* = 0.001). For LAD, the HNCM group showed a significant increase compared to the control group (*p* = 0.0270), but there was no marked enlargement of the left atrial diameter across the entire HCM group (LAD < 16 mm). In cases with fusion waves, there were six cats in the control group, two cats in HNCM group and six cats in HOCM group. There were no significant differences in Ev between the three groups, but e’ FW was significantly reduced in both the HOCM and HNCM groups compared to the control group (*p* = 0.0243, 0.0455), and E/e’ FW was significantly increased (*p* = 0.0385, 0.0021). No significant differences were observed between the HOCM and HNCM groups, except for LVOTv (*p* = 0.0076).

### 3.3. Color M-Mode Echocardiography for IVPG

**Figure 3:**
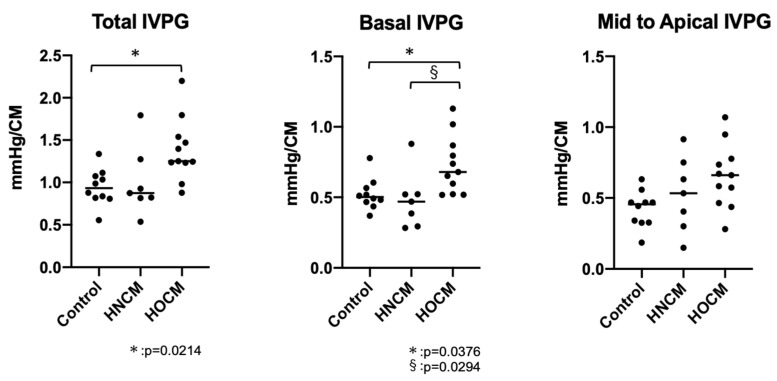
CMM-derived intraventricular gradients in the control (*n* = 10), HNCM (*n* = 7) and HOCM (*n* = 11) groups. Horizontal lines indicate median values. *, §, indicates statistical significance (*p* < 0.05). HNCM: hypertrophic non-obstructive cardiomyopathy; HOCM: hypertrophic obstructive cardiomyopathy; IVPG: intraventricular pressure gradient. Total IVPG and basal IVPG were significantly increased in the HOCM group compared to the control group. Basal IVPG was significantly higher in the HOCM group compared to the HNCM group. No differences in mid-to-apical IVPG were observed between the groups.

In this study, IVPG analysis was not possible in three cats with HOCM due to the presence of severe left ventricular outflow tract (LVOT) obstruction. In total, IVPG analysis was successful in 25 of 28 cases, with an analysis rate of approximately 89.3%. However, data from the three cats that could not be analyzed were able to be analyzed after treatment and were included in this study. Previous reports on healthy cats showed a correlation between Ev and IVPG [12]; however, in this study, no significant correlation was found between Ev and basal IVPG (*p* = 0.0072, rc = 0.496). Additionally, there was no correlation between total IVPG and mid-to-apical IVPG with Ev (rc = 0.418, 0.310). The HOCM group showed a significant increase in total IVPG (*p* = 0.0214) and basal IVPG (*p* = 0.0376) compared to the control group (Figure 3, Table 2). Basal IVPG was significantly higher in the HOCM group compared to the HNCM group (*p* = 0.0294).

## 4. Discussion

### 4.1. Significance of IVPG in Assessing Diastolic Function and Clinical Applications in Veterinary Cardiology

IVPD and IVPG can help clarify conditions that conventional echocardiography alone cannot explain, making them excellent indicators of diastolic function [12]. It is believed that IVPD is influenced not only by volume load and diastolic function but also by the size of the heart and age. However, IVPG is adjusted by the left ventricular long-axis length, making it less affected by heart size [4,13]. In veterinary medicine, IVPG is still in the experimental stage, but our study using dogs with PDA (patent ductus arteriosus) demonstrated that IVPG responds more consistently to preload reduction than to left ventricular relaxation. This result supports the clinical utility of CMM-derived IVPG measurements in assessing diastolic function in dogs with PDA at an early stage [14]. Because heart morphology among dog breeds is relatively similar, with adequately sized intracardiac cavities, dogs are well-suited for IVPG measurements. In diseases like PDA, where the intracardiac cavity is further enlarged, IVPG measurements become even easier. In contrast, cats have smaller intracardiac cavities, making IVPG measurements more challenging than in dogs. However, we have measured IVPG in 106 healthy cats, demonstrating that IVPG measurement is feasible in cats and revealing that it is more susceptible to increased heart rate and a short IVRT (isovolumetric relaxation time) [15]. In this process, the need for future research to evaluate segmental IVPG changes in cats with HCM became evident, particularly to clarify how CMM-estimated IVPG can be applied clinically in feline cardiology. This study aims to demonstrate the clinical utility of IVPG in HCM cases and to further elucidate the impact of left ventricular outflow tract obstruction on IVPG by classifying HCM into HOCM and HNCM.

Conventional echocardiographic parameters like TDI (specifically, e’ and E/e’ ratios) have long been employed to assess diastolic function, with increased E/e’ values indicating elevated left ventricular filling pressures and compromised relaxation [16]. However, as noted, the high heart rates often observed in feline HCM cases can obscure mitral inflow patterns due to fusion of the E and A waves, making accurate diastolic evaluation challenging [17]. IVPG, on the other hand, is a diastolic indicator that remains relatively unaffected by heart size and rate, thus allowing for a reliable assessment even under these conditions. By integrating IVPG with conventional diastolic markers, we can achieve a more comprehensive understanding of diastolic impairment and LA pressure changes, particularly in cases with LVOT obstruction, where IVPG elevation may indicate hemodynamic instability that conventional metrics alone might not detect.

There are several limitations to the analysis of IVPD and IVPG using CMM. Firstly, arrhythmias disrupt the cardiac cycle, impeding normal myocardial relaxation, and thereby making consistent analysis difficult. Secondly, in cases of aortic valve regurgitation, diastolic blood flow enters the left ventricle and collides with mitral inflow, rendering accurate evaluation challenging. Thirdly, severe mitral regurgitation or turbulence at the apex can distort CMM imaging, significantly complicating the analysis of blood flow dynamics. These factors pose substantial challenges to reliable IVPD and IVPG measurement using CMM.

### 4.2. Characteristics of the Study Group and Differences in Echocardiographic Features Between HOCM and HNCM

This study included 18 cats (11 with HOCM, 7 with HNCM) and 10 control cats. The relatively small sample size and the inability to match the age and body weight distribution between groups are limitations. The IVPG analysis was conducted by inputting CMM image data into a MATLAB analysis program. However, in cases where analysis was not feasible, errors occurred. In some cases, in this study, the analysis could not be performed. All of these unmeasurable cases were HOCM cases, which had severe congestion or significant LVOT obstruction and turbulence near the apex of the heart. For these cases, data from earlier stages of the disease, when the condition was milder, were used, meaning that IVPG data for more severe conditions were not reflected. The fact that measurement is impossible in severe cases poses a significant problem for the examination method, which must be addressed. In cases where the heart rate increased due to excitement, special care was required to calm the animal during the echocardiographic examination [15].

Previous studies on healthy cats have also shown that heart rate affects IVPG, underscoring the importance of conducting exams in a more natural state to obtain reliable data, regardless of whether analysis is possible.

In this study group, both IVSd and LVPWd were elevated in both the HOCM and HNCM groups, indicating myocardial hypertrophy in both groups. The measurement of e’ using TDI is relatively independent of volume load [18,19], and the decrease in e’ observed in both the HOCM group and HNCM group in this study suggests left ventricular relaxation impairment or decreased myocardial flexibility in HCM.

However, there was no difference in FS, and there were no cases of marked left atrial enlargement in terms of LAD. There were no significant differences in the E wave between the groups, suggesting that the left atrial pressure elevation was not severe. Although the increase in the E/e’ ratio implies an elevation in left ventricular end-diastolic pressure (LVEDP) [16,20,21], and hence an increase in left atrial pressure, the E/e’ values were not significantly higher than normal, and as no morphological changes like atrial enlargement were observed, it can be concluded that the congestion was mild. According to conventional echocardiography findings, both the HOCM and HNCM groups exhibit left ventricular relaxation dysfunction or reduced myocardial flexibility due to myocardial hypertrophy, with mild congestion, and no significant differences were observed between the two groups. While the difference in LVOT velocities allows for easy differentiation between HOCM and HNCM using conventional echocardiography, it is interesting that there were no differences between the groups in terms of disease severity. Kizilbash et al. reported that a single measurement of the left ventricular outflow tract is insufficient to define the severity of dynamic left ventricular outflow tract obstruction [22]. In reports on cats, it has been noted that the use of pimobendan in HOCM does not cause sudden hemodynamic changes [23]. However, in cases with SAM, the use of pimobendan has been reported to worsen the condition due to hypotension [24]. This suggests that useful information for treatment selection may not be obtained. In HNCM, there is no outflow tract obstruction, so the primary pathology is impaired diastolic function. On the other hand, in HOCM, the degree of LVOT obstruction may vary during the cardiac cycle, potentially influencing the internal environment of the left ventricle. Conventional echocardiography may not be useful in assessing this difference.

### 4.3. Is Basal Congestion?

By dividing IVPG into three segments, it becomes possible to categorize pathological conditions in greater detail. Basal IVPG, in particular, is thought to correlate with left atrial pressure [25]. In this study, basal IVPD was elevated exclusively in the HOCM group in contrast to the control group. This finding suggests that evaluating heart function in feline HOCM may be challenging based solely on left atrial enlargement. At the same time, it highlights the importance of basal IVPG as a key indicator in assessing congestion in feline HOCM.

In HOCM, the hypertrophy of the interventricular septum narrows the left ventricular outflow tract, creating resistance to blood flow from the left ventricle to the aorta. This obstruction increases blood flow velocity near the left ventricular base (around the outflow tract), which likely leads to an elevation in IVPG. Particularly during systole, a significant pressure difference between the left ventricle and the aorta is generated, contributing to the rise in basal IVPG. The dynamic obstruction characteristic of HOCM, where sudden worsening occurs during certain parts of the cardiac cycle, may also result in a marked increase in IVPG at the ventricular base, which is likely reflected in this study.

Furthermore, in HOCM, a phenomenon called SAM often occurs, where the anterior mitral valve leaflet is pulled into the left ventricular outflow tract. This changes the direction of blood flow at the ventricular base, causing a sharp increase in flow velocity, which likely leads to a further elevation in basal IVPG. Additionally, mitral regurgitation caused by SAM can lead to an increase in left atrial pressure and volume overload in the left ventricle. This may raise the pressure on the basal side of the heart, potentially causing an increase in basal IVPG.

Given that the severity of HOCM and HNCM in this study is considered mild, the elevation of basal IVPG observed only in the HOCM group should be understood as being caused by factors other than congestion or increased left atrial pressure. In studies on canine patent ductus arteriosus (PDA), basal IVPG was a good parameter for assessing congestion, but in cats, particularly those with HOCM, basal IVPG cannot be considered a highly sensitive indicator of congestion. It is important to recognize that various effects associated with left ventricular outflow tract obstruction may also cause elevated basal IVPG in these cases.

### 4.4. Differences in IVPG Between HNCM and HOCM

Caution is also needed when interpreting IVPG other than basal IVPG. In our previous animal experiments, it was shown that an increase in volume load leads to an increase in basal IVPG, while a decrease in diastolic function causes a reduction in mid-to-apical IVPG [6,11]. A similar analysis was conducted in this study, but significant increases were observed only in the HOCM group. In this group, total and basal IVPG showed an increase.

In HNCM, the main pathology was believed to be impaired diastolic function, as there is no outflow tract obstruction. We hoped that using IVPD would allow for a detailed assessment of the pressure gradient within the left ventricle, helping us evaluate relaxation dysfunction and abnormal diastolic dynamics. However, unfortunately, we could not find any significant differences compared to the control group. This suggests that using IVPD alone may not be sufficient to evaluate the pathophysiology of HNCM.

On the other hand, the observed increases in total and basal IVPG in the HOCM group also require careful consideration. As mentioned earlier, the rise in basal IVPG is likely due to the factors previously discussed, but as basal IVPG is part of the total and mid-to-apical IVPG measurements, the increase in these areas may also be influenced by the elevated basal IVPG. In studies on humans and dogs, the heart is divided into three segments, and only basal IVPG near the atrium is considered to be influenced by left atrial pressure. However, given that the feline heart is smaller and more compact, it is possible that left atrial pressure affects not only basal IVPG but also mid and apical IVPG. Therefore, it may be necessary to reconsider the method of segmenting IVPG when applied to cats.

### 4.5. The Significance of IVPG in Diastolic Function in Cats

In this study, we aimed to assess the potential of IVPG as a novel marker for diastolic function in cats with HCM, particularly in differentiating HOCM from HNCM. Unlike conventional echocardiographic metrics such as the E/A ratio and TDI, IVPG offers distinct advantages, especially in cases of elevated heart rates often observed in cats, where the fusion of diastolic waves can obscure accurate evaluation of mitral inflow patterns [17]. IVPG derived from color M-mode echocardiography remains relatively unaffected by heart size and rate, allowing consistent assessment of diastolic function dynamics even under challenging clinical conditions [15]. This is particularly relevant in HOCM, where LVOTO may introduce significant pressure variations within the ventricle, influencing hemodynamic stability and impacting overall cardiac function [9]. By segmentally assessing IVPG along the left ventricle, this study seeks to highlight how IVPG can better reflect the intraventricular pressure changes associated with LVOTO in HOCM, potentially guiding future clinical management of diastolic dysfunction in feline cardiology. Thus, our research is designed to expand the understanding of IVPG’s clinical applicability, offering a more nuanced approach to evaluating HCM cases in veterinary practice.

Previous studies, including those by Spirito et al. and Maron et al., have shown that high IVPG is associated with hemodynamic instability and poor prognosis in cases with outflow tract obstruction, linking it to an increased risk of syncope, heart failure, and sudden death [26,27]. Our findings align with these reports, as basal IVPG was notably elevated in the HOCM group, likely reflecting LVOTO-related pressure dynamics. This suggests that basal IVPG may serve as a valuable indicator of diastolic dysfunction and hemodynamic changes under obstruction. We believe that wider clinical use of IVPG, especially in assessing LVOTO in feline cardiology, could enhance prognosis management, warranting further clinical trials [9,28].

## 5. Limitations

Although the number of cats included in this study is small, the data provided, and their clinical relevance are solid. In addition, the age of the patients was relatively young, and treatment varied from patient to patient. Finally, we did not perform a comparative evaluation of CMM and catheterized IVPD in the present study, as previous studies have shown that CMM-derived IVPD and IVPG correlate strongly with tau measured by invasive catheterization [10,29].

## 6. Conclusions

Traditional echocardiographic indicators have primarily focused on morphological perspectives and blood flow velocity. In this study, conventional echocardiography did not reveal differences in pathophysiology between the HOCM and HNCM groups. However, by using IVPG for the evaluation, increases in total and basal IVPG in the HOCM group were detectable. While HOCM and HNCM can be easily differentiated by LVOT velocity, considering the mechanism of IVPG elevation in HOCM, IVPG may better reflect the condition. The pathophysiology of HOCM is influenced by many factors, making its assessment challenging. Traditional echocardiographic indicators require comprehensive knowledge and experience to evaluate the combination of various metrics. However, IVPG offers a simpler and more valuable method for evaluating the impact of left ventricular outflow tract obstruction on the condition.

## Figures and Tables

**Table 1:** Conventional echocardiographic indices for control and HCM groups.

					*p* Value	
	Control (*n* = 10)	HNCM (*n* = 7)	HOCM (*n* = 11)	Control vs. HNCM	Control vs. HOCM	HNCM vs. HOCM
IVSd (mm)	4.4 (2.7–5.7)	6.0 (4.5–8)	6.2 (4.9–7.5)	0.0082 *	0.0013 *	>0.9999
LVPWd (mm)	3.7 (2.6–4.6)	6.1 (4.2–7.4)	6.2 (4.3–6.8)	0.0024 *	0.001 *	>0.9999
HR (bpm)	184.5 (121–198)	171 (151–210.7)	155.7 (122–180)	>0.9999	0.1474	0.2133
FS (%)	47.2 (36.2–61.1)	53.8 (43.5–81.5)	53.8 (44.3–75.4)	0.966	0.3115	>0.9999
LA/Ao	1.3 (1.2–1.6)	1.4 (1.2–1.9)	1.45 (1.2–1.8)	0.3993	0.0402 *	>0.9999
LAD (mm)	11.3 (10–13.3)	13.7 (10.6–14.3)	12.4 (8.7–13.9)	0.027 *	0.3813	0.5978
LVOTv (cm/s)	80.0 (64.6–114.8)	108.0 (66.4–157.6)	446.5 (221.4–593.6)	>0.9999	<0.0001	0.0076 *
Ev (cm/s)	86.2 (53.5–113.7)	83.1 (59.2–108.6)	95.3 (50.1–141.0)	>0.9999	0.3541	0.5613
s’ FW (cm/s)	5.9 (4.4–8.8)	5.7 (3.4–6.5)	6.2 (3.6–13)	0.7984	>0.9999	0.79
e’ FW (cm/s)	9.3 (5.3–14.4)	6.1 (4.4–8.7)	5.8 (4.3–10.6)	0.045 *	0.0243 *	>0.9999
E/e’ FW	7.7 (2.8–12.2)	12.8 (6.9–18.9)	12.8 (6.9–18.9)	0.0385 *	0.0021 *	>0.9999

Values are presented as median (data range). *, indicates statistical significance (*p* < 0.05). IVSd: interventricular septum thickness at end-diastole; LVPWd: left ventricular posterior wall thickness at end-diastole; HR: heart rate; FS: left ventricular fractional shortening; LA/Ao: left-atrial-to-aortic ratio; LAD: left atrial diameter; LVOTv: left ventricle outflow tract velocity; E: peak velocity of early diastolic transmitral flow velocity; s′: peak velocity of systolic mitral annular motion as determined by color-based tissue Doppler imaging; e’: peak velocity of early diastolic mitral annular motion as determined by color-based tissue Doppler imaging; FW: free wall.

**Table 2:** Comparison of each IVPG by CMM among control and HCM groups.

					*p* Value	
	Control (*n* = 10)	HNCM (*n* = 7)	HOCM (*n* = 11)	Control vs. HNCM	Control vs. HOCM	HNCM vs. HOCM
Total IVPG (mmHg/cm)	0.94 ± 0.21	0.93 ± 0.41	1.25 ± 0.68	>0.9999	0.0214 *	0.0592
Basal IVPG (mmHg/cm)	0.52 ± 0.11	0.50 ± 0.20	0.68 ± 0.31	>0.9999	0.0376 *	0.0294 *
Mid-to-apical IVPG (mmHg/cm)	0.42 ± 0.13	0.46 ± 0.26	0.66 ± 0.40	>0.9999	0.0703	0.7593
Mid IVPG (mmHg/cm)	0.32 ± 0.08	0.33 ± 0.21	0.52 ± 0.30	>0.9999	0.3879	0.7135
Apical IVPG (mmHg/cm)	0.10 ± 0.06	0.08 ± 0.11	0.15 ± 0.13	>0.9999	0.8258	>0.9999

Values are presented as median ± standard deviation. *, indicates statistical significance (*p* < 0.05). IVPG: intraventricular pressure gradients; HNCM: hypertrophic non-obstructive cardiomyopathy; HOCM: hypertrophic obstructive cardiomyopathy.

## Data Availability

The original contributions presented in the study are included in the article, further inquiries can be directed to the corresponding author.
